# Hands off Trans-Femoral Venous Intra-Abdominal Pressure Estimates in Children: Results of a Sobering Single-Center Study

**DOI:** 10.3390/life13040872

**Published:** 2023-03-24

**Authors:** Miriam Gutting, Lara Klischke, Torsten Kaussen

**Affiliations:** Department of Pediatric Cardiology and Intensive Care Medicine, Hannover Medical School, Carl-Neuberg-Str. 1, D-30625 Hannover, Germany

**Keywords:** femoral venous pressure, intra-abdominal pressure, intra-vesical pressure, intra-gastral pressure, intra-abdominal hypertension, children, pediatric

## Abstract

Background: For a long time, trans-femoral venous pressure (FVP) measurement was considered a simple alternative for estimating intra-abdominal pressure (IAP). Since intravesical [IVP] and intragastric [IGP] pressure measurements are sometimes contraindicated for anatomical and pathophysiological reasons, FVP raised hopes, especially among pediatricians. Pediatric FVP validation studies have never been published; recent results from adult studies cast doubt on their interchangeability. Therefore, we compared for the first time the measurement agreement between FVP and IVP and IGP in children. Material and methods: We prospectively compared FVP with IVP and IGP, according to the Abdominal Compartment Society validation criteria. Additionally, we analyzed the agreement as a function of IAP or right heart valve regurgitation and pulmonary hypertension. Results: In a real-life PICU study design, n = 39 children were included (median age 4.8 y, LOS-PICU 23 days, PRISM III score 11). In n = 660 FVP–IGP measurement pairs, the median IAP was 7 (range 1 to 23) mmHg; in n = 459 FVP–IVP measurement pairs, the median IAP was 6 (range 1to 16) mmHg. The measurement agreement was extremely low with both established methods (FVP–IGP: r^2^ 0.13, mean bias −0.8 ± 4.4 mmHg, limits of agreement (LOA) −9.6/+8.0, percentage error (PE) 55%; FVP–IVP: r^2^ 0.14, bias +0.5 ± 4.2 mmHg, limit of agreement (LOA) −7.9/+8.9, percentage error (PE) 51%). No effect of the a priori defined influencing factors on the measurement agreement could be demonstrated. Conclusions: In a study cohort with a high proportion of critically ill children suffering from IAH, FVP did not agree reliably with either IVP or IGP. Its clinical use in critically ill children must therefore be strongly discouraged.

## 1. Introduction

The measurement of intra-abdominal pressure (IAP) is an important factor for the detection of abdominal compartment syndrome (ACS) in critical ill adults and children [1,2,3]. Early recognition and treatment are essential for prognosis and outcome [4,5]. In children, a consistently intra-abdominal hypertension (IAP > 10 mmHg) in combination with acute or worsening organ dysfunction attributed to the elevated IAP is defined as ACS [6]. 

There are different methods to measure IAP. The Abdominal Compartment Society (WSACS) currently advises bladder pressure measurement (synonym: intravesical pressure measurement (IVP)) as the benchmark technique for quantifying intra-abdominal pressure in non-adults [6]. The intra-vesical measurement is susceptible to disruptive factors such as patient positioning changes or artifact formation due to the fluid-filled system. A further disadvantage is that the measurements take place at certain points in time and not continuously, so that an acute increase in IAP could possibly be overlooked. In fact, regular the measurement of IAP is rather rare in clinical intensive care units [5,7,8].

Another option to evaluate IAP is the measurement of intra-gastric pressure via a nasogastric tube (IGP), which allows a continuous monitoring with similar susceptibility to faults. Recently, our research group validated a continuous intra-gastric measurement system for children and adolescents [9].

Transfemoral venous pressure (FVP) has been postulated for years as a measurement equivalent and trend monitor to assess IAP values [10,11,12,13]. In particular in preterm, neonates, babies and toddlers, the method was considered practicable, elegant and promising because the anatomy, pathophysiology and body size ratios of small children often make the use of established measurement methods via the bladder or the stomach (IVP, IGP) impossible or too risky. Nevertheless, based on study results from adult medicine, there has been increasing evidence in recent years that the measurement accuracy, sensitivity and reliability of the FVP method leave much to be desired. Remarkably, no form of FVP validation or usability studies have ever been performed in pediatrics and adolescent medicine.

Therefore, we conducted a single-center study to evaluate the reliability of the FVP measurement in relation to the detection of increased IAP. For this purpose, FVP measurements were compared with concurrent IVP (standard procedure) and IGP measurements in critically ill children and adolescents in a supraregional university pediatric intensive care unit (PICU), and the strength of the agreement was quantified.

## 2. Materials and Methods

### 2.1. Design of the Study

The study site of this prospective observational study—realized between January 2015 and February 2017—was the interdisciplinary pediatric intensive care unit of Hannover Medical School (MHH). The single-center clinical trial was authorized by the MHH local ethics board (ID 6677) and registered worldwide (ICTRP: DRKS00006556). 

### 2.2. Patient Study Recruitment

All neonatal and pediatric patients who needed an intra-abdominal pressure (IAP) measurement via IVP or IGP and additionally a femoral central venous catheter were enrolled in the study. If the children did not need a stomach tube and/or a bladder catheter or a femoral central line due to their underlying disease, no devices were placed for study purposes; inclusion in the study was therefore not conceivable in such cases. The exclusion criteria comprised prematurity or immaturity at birth as well as any form of malformation, injury or other diseases of the mouth, pharynx, esophagus and stomach or urogenital tract that could make the insertion of a nasogastric tube or bladder catheter (or a catheter equivalent) difficult, dangerous or impossible. Prior to study participation, written informed consent was requested from the patients and/or their guardians.

### 2.3. Clinical Data Collection

In addition to the different measurement methods of IAP, defined biographical, anthropometric and prognostic data were collected and documented for each enrolled subject. These included, in particular, the diagnoses justifying admission, the duration of the stay at PICU (LOS-PICU) and the Pediatric Risk of Mortality III Score (PRISM III) [14]. This should facilitate the unmasking of possible influences on the agreement between the different measurement methods. 

### 2.4. Intra-Abdominal Pressure Measurement (IAP)

Intra-abdominal hypertension (IAH) was defined as IAP > 10 mmHg in at least two consecutive IAP measurements, an abdominal compartment syndrome (ACS) as IAH accompanied by organ dysfunction (new or deteriorating) [6]. According to child-adapted WSACS definitions [5], IAH was classified into four grades (I°: IAP >10–12 mmHg, II°: 13–15 mmHg, III°: 16–18 mmHg, IV°: >18 mmHg). 

### 2.5. Measurement Practice

Based upon the modified Kron technique [9,15], IVP measurements were performed using a transurethral catheter according to WSACS recommendations (emptying the bladder, filling with 1 mL/kg of BW normal saline (min. 3 mL, max. 25 mL) under aseptic conditions, waiting for at least 2 min to allow equilibration) with the midaxillary level as the zero reference (clinical standard). IVP is transmitted from the end-open transurethral catheter through the continuous liquid column in the catheter lumen to an outside pressure transducer (Codan, Germany). Transurethral catheters for IVP measurement were sizewise adjusted for weight and age (Norta-Nelaton 6–16 Charriére (Ch.) diameter, BSNmedical Company, Germany). For anatomical reasons, gastric tubes were used alternatively in small neonates (Flocare pursoft tube, 5 Ch., Nutricia Medical Devices, The Netherlands).

IGP was determined by air capsule-based measurement (Spiegelberg company, Germany) using a commercially available 9 French double-lumen nasogastric tube catheter with one lumen for continuous IGP measurement and another for regular feeding. The accurate intragastric or intravesical placement of the IGP and transurethral catheters was verified by ultrasound at minimum once a day and, in addition, whenever the IGP or IVP measurements did not reveal respiratory undulations [9].

The femoral vein pressure (FVP) was measured via the fluid-filled and flushed (1 mL/h), distal limb of a three-lumen central venous catheter (CVC) inserted into the femoral vein using the Seldinger technique (Vygon Multicath-3 Pädiatrie, Stolberg, Germany), with a pressure transducer and an A/D converter on the patient-monitoring unit from GE (General Electrics).

To assess the influence of possible tricuspid and/or pulmonary valve leakage on the FVP, an experienced pediatric cardiologist examined the subjects at least once by echocardiography and estimated the pulmonary arterial pressure that could be derived from the right cardiac valve insufficiency gradients [16].

### 2.6. Agreement of FVP with IVP/IGP

Simultaneous IAP readings were taken in all patients every hour during the day to assess compliance and to perform exploratory analyses to evaluate the possible effect of predefined predictive factors. The data were recorded either up to discharge from the pediatric intensive care unit or up to withdrawal of the IGP/IVP and FVP catheters, whichever occurred earlier. To avoid artefact-related false measurements, pressure measurements during the weaning of recovering patients were only used and analyzed if the patients did not show signs of agitation with or without mass movements but had stable and age-appropriate monitoring vital signs. Furthermore, IAP measures obtained during physician visits, nursing care and procedures, rehabilitative therapies and other examinations or procedures were eliminated and neither analyzed nor scored.

### 2.7. Explorative Analysis of Confounders

To identify possible factors influencing the robustness of the FVP measurements, the agreement of the measurement results depending on right ventricular valve insufficiencies and pulmonary arterial pressures on the one hand and on different IAP heights on the other hand was investigated in a second step in an explorative analysis.

(1) Right heart evaluation was performed by an experienced pediatric cardiologist using a Philips echocardiography device (CX50, Koningklijke Philips N.V, Eindhoven, The Netherlands). For the classification of valve insufficiencies into mild, moderate and severe forms, the definitions according to the guidelines and recommendations of the American (2017) [17] and European (2021) [18] professional societies for cardiology and echocardiography, respectively, were applied. According to the Nice Criteria of 2018, pulmonary hypertension (PHT) was defined in the case of an echocardiographically assessable mean pulmonary arterial pressure (mPAP) of mPAP > 20 mmHg [16].

The results were divided into 4 groups:

- Group 1: no tricuspid valve (TI) and/or no pulmonary valve insufficiency/regurgitation (PI) and no pulmonary hypertension (PHT).
- Group 2: mild TI and/or mild PI and no PHT
- Group 3: mild to moderate TI and/or mild to moderate PI and signs of PHT
- Group 4: no echocardiographic results available
- No child showed signs of severe TI and/or PI.

(2) Depending on the IAP measured via IVP or IGP, the results were grouped into IAP classes:

- Group 1: IAP < 7 mmHg (corresponds to normal IAP in children)
- Group 2: IAP 7–9 mmHg (corresponds to “pre-IAH” especially in neonates and infants)
- Group 3: IAP 10–12 mmHg (corresponds to IAH grade I in children)
- Group 4: IAP 13–15 mmHg (corresponds to IAH grade II in children)
- Group 5: IAP 16–18 mmHg (corresponds to IAH grade III in children)
- Group 6: IAP > 16 mmHg (corresponds to IAH grade IV in children)

### 2.8. Data Processing and Statistical Analysis

Clinical data were collected using a digital patient data monitoring system (Copra^®^Systems, Berlin, Germany) and transferred to Excel^®^2016 (Microsoft^®^ Corporation Redmond, WA, USA). The statistical analysis was performed using SPSS^®^ Statistics 22.0 (IBM^®^, Armonk, North Castle, Armonk, NY, USA). The results of the simultaneous IAP readings were juxtaposed by means of a linear regression analysis. As there was no normal distribution (as per Shapiro–Wilk testing), the correlation analysis was carried out applying Spearman’s coefficient of determination. Following the WSACS guidelines (Table 1), the agreement and thus the exchangeability of the IAP assessment techniques was judged according to Bland–Altman, whereby the so-called precision corresponds to the standard deviation (SD) of the mean bias, and the percentage error (PE) to the quotient of limits of agreement (LOA) and the mean value of the IAP measured with both techniques at the same time [19]. Furthermore, a mean absolute percentage error (MAPE ± SD) was calculated according to de Myttenaere et al. [20]. The 1988 Cohen criteria were applied to interpret the Spearman’s correlation coefficients [21].

## 3. Results

### 3.1. Patients Characteristics

Two groups, each comprising *n* = 19 (IVP group) and *n* = 20 patients (IGP group), were set up to compare the FVP with the reference method of IVP or IGP, respectively. Sex distribution (32% and 35% female) and average age, with a median of 4.9 years in the IVP group and 4.7 years in the IGP group, were almost identical (Table 2). In the cohort of the IGP measurement, with *n* = 660, the number of paired measurements was higher compared to the number of paired FVP measurements in the IVP cohort (*n* = 459). To illustrate the extensive homogeneity of both subgroups, the results of the descriptive statistics are summarized in Table 2. The PRISM III score, as an indicator for mortality in pediatric intensive care units, reflected similar disease severity in both groups at the beginning and end of the observation. One patient in the IGP group died.

### 3.2. Comparison between FVP and IGP

Table 3 shows the results for the comparison between FVP and IGP measurements. 

#### 3.2.1. Overall Results

**Figure 1:**
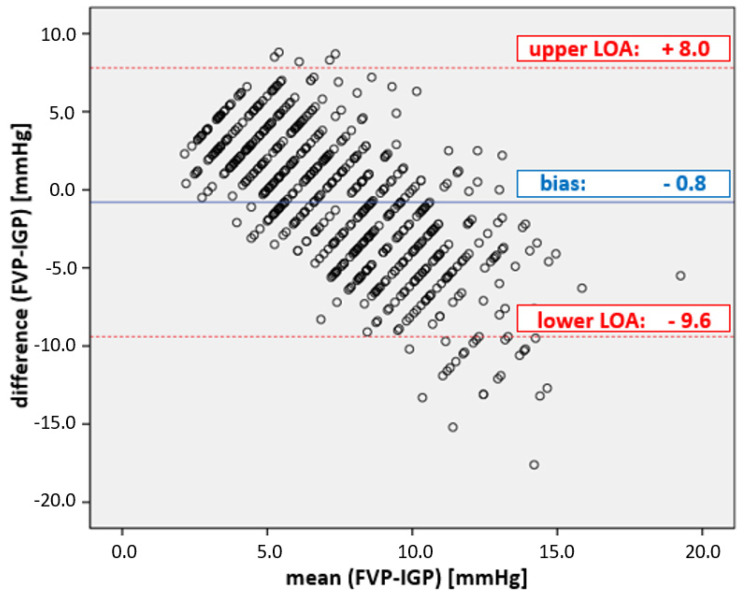
Plot of the agreement between FVP and IGP measurements. Bland–Altman diagram of FVP and IGP. The mean bias ± precision between FVP and IGP was −0.8 ± 4.4 mmHg; the limits of agreement (LOA) were from −9.6 to + 8.0 mmHg, well outside the acceptable boundaries set by WSACS (Table 1).

With a correlation coefficient of r^2^ = 0.13, the overall agreement between IGP and FVP measurements was very low. While the WSACS-defined compliance criteria for alternative measurement methods (Table 1) were met for bias (−0.8 mmHg), they were far outside the tolerable range for precision (with 4.4 mmHg) and limits of agreement (with results between −9.6 and 8.0 mmHg; see Figure 1). Furthermore, the percentage error was 117%, clearly above the cut-off.

#### 3.2.2. Explorative Additional Examinations

In the context of the explorative analysis of potential influencing factors, only children with elevated pulmonary arterial pressure showed a slightly improved correlation result according to Spearman, with r^2^ = 0.47 in this subgroup. In children without PHT, the Spearman’s correlation coefficient varied between just r^2^ = 0.1 and no more than r^2^ = 0.13, depending on the presence of right ventricular insufficiency (see Table 3). 

A comparably poor agreement between r^2^ = 0.1 and r^2^ = 0.15 was also found for all IAP- and IAH-grade-dependent subgroup analyses (see Table 3). All other agreement criteria were far from being met in all subgroups (incl. PHT) (bias, precision, limits of agreement, percentage error).

### 3.3. Comparison between FVP and IVP

Table 4 shows the comparison between IVP and FVP measurements. 

#### 3.3.1. Overall Results

**Figure 2:**
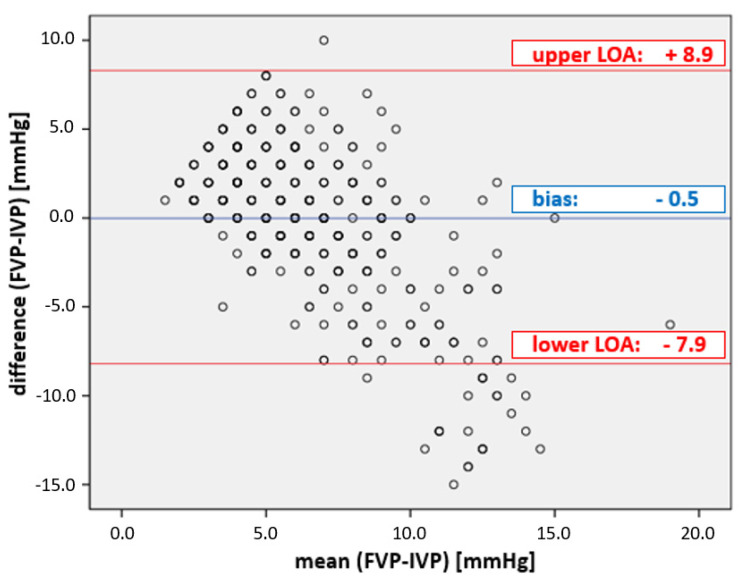
Plot of the agreement between FVP and IVP measurements. Bland–Altman diagram of FVP and IVP. The mean bias ± precision between FVP and IVP was −0.5 ± 4.2 mmHg; the limits of agreement (LOA) were from −7.9 to + 8.9 mmHg, well outside the acceptable boundaries set by WSACS (Table 1).

The overall correlation between IGP and FVP measurements was very low (r^2^ = 0.14). While the bias of −0.5 mmHg was in the given range (Table 1), the precision of 4.2 mmHg and the limits of agreement from −7.9 to 8.9 mmHg did not fulfill the given limits of the criteria (see Figure 2); the percentage error was 133%, clearly above the cut-off (25%). 

#### 3.3.2. Exploratory Additional Examinations

In the context of the exploratory analysis of potential influencing factors, children with valvular insufficiencies or increased pulmonary arterial pressure showed a slightly better correlation result according to Spearman, with r^2^ = 0.3 and r^2^ = 0.47, respectively (Table 4), whereas children without right ventricular insufficiencies showed low correlations, from just r^2^ = 0.02 to r^2^ = 0.04 (see Table 4). 

Similarly low correlations were found in the IAP- or IAH-grade-dependent subgroup analyses (Table 4). Only children with IVth-grade IAH (IAP > 18 mmHg) showed a stronger correlation, with r^2^ = 0.68.

All other WSACS agreement criteria for alternative measurement methods (Table 1: bias, precision, limits of agreement, percentage error) were far from achieved in all subgroups (incl. PHT and IAH grade IV) [Table 4].

## 4. Discussion

### 4.1. Study Design and Key Messages

After the reliability of FVP quantification—proposed as an alternative IAP measurement method in the 1980s and 1990s (Table 5)—had already been questioned by studies on adults, the main aim of the present monocentric study was to test the FVP measurement methodology for the very first time in children. For this reason, we performed a prospective cross-sectional validity evaluation to assess this IAP quantification technique in *n* = 39 children with critical illness. Remarkably, for the first time, this validation study fulfilled without exception all criteria postulated by the WSACS for the comparison of different IAP measurement methods in a clinical real-life pediatric ICU setting. We were able to collect reliable data across a broad pediatric age spectrum with a representative disease severity and distribution (Table 1). Our study cohort is of particular value in terms of its validation power based on the frequent occurrence of elevated abdominal pressures (IAH), ranging from 22% (IVP group) to 43% (IGP group) (i.e., cases with IAH grade III (16–18 mmHg) and IV (>16 mmHg) and IAP heights of up to 23 mmHg).

In this challenging population, we proved beyond doubt that IAP estimation by FVP quantification is useless in everyday clinical practice in the PICU. It is neither accurate, nor precise, reproducible or reliable against the actual medical benchmark method, bladder pressure measurement (IVP) and the validated measurement method alternative, namely, intra-gastric pressure measurement (IGP).

In an additional exploratory analysis, we also investigated clinical factors such as right cardiac valvular regurgitation and/or increased pulmonary arterial pressure (PHT), as well as IAP level per se, each of which could affect the agreement of the IAP measurement. Overall, we found neither positive nor negative influences of these potential confounders on the measurement agreement of FVP. Even if a somewhat stronger Spearman’s correlation was achieved in the presence of a PHT or with higher-grade IAH (max. r^2^ = 0.68), the other WSACS-postulated agreement criteria were still far from being achieved (see bias, precision, limits of agreement and percentage error, as shown in Table 3 and Table 4).

Based on these observations, the use of the FVP as a method for estimating the IAP in children must be strongly discouraged.

### 4.2. Medical Historical Motivations for This IAP Estimation Validation Study

In 1987, Lacey’s research group pioneered the idea of utilizing the trans-femoral venous pressure (FVP) for IAP estimation in neonates suffering from abdominal wall defects [22]. In an animal model, they compared the intraoperative agreement of different indirect IAP pressure measurement methods in 17 rabbits. The pressures measured intra-vesically and trans-femorally in the inferior vena cava (IVC) agreed best with the applied IAPs. In 2002, Gudmundson et al. wanted to verify the results in a large animal model and also found a strong correlation between IAP and FVP or IVP in eight pigs [11]. However, the applied pressure level of 15–40 mmHg was clearly above the IAP level that is regularly reached in clinical practice. When Jakob et al. [23] took this into account and tested the measurement agreement in 13 pigs in a more clinically relevant range between 0 and 22 mmHg, the first doubts arose about the clinical relevance and applicability of the trans-femoral measurement method, which appeared quite attractive due to its potentially continuous measurement methodology. In their pig study published in 2011, Regli et al. increasingly applied the agreement criteria for alternative IAP measurement methods defined by the WSACS and in particular objectified a far too large range with regard to the limits of agreement [24]. 

This temporal development from initial recommendation to interim doubt to final rejection of sufficient agreement and significance was also shown with a few years’ latency in the context of human clinical studies [12,13,25,26] (Table 5). The more the WSACS postulated criteria for agreement were applied over time, the more objective and clear the discrepancy between the alternative methods became. The working groups around De Keulenaer [25] and Howard [26] finally made the inadequacy of the FVP unmistakably clear. 

Although the initial impetus for FVP measurement came from neonatal pediatric surgeons [22], any clinical translation and verification in a pediatric intensive care setting has been lacking. We wanted to take this into account and close the evidence gap that existed until then.

### 4.3. Clinical Implications

FVP has also proven to be useless in pediatric intensive care. IAP monitoring in the sensitive pediatric setting should therefore be limited to the established and validated methods of bladder and gastric pressure measurement until further notice [9]. Nonetheless, any increase in trans-femoral venous pressure should, of course, suggest the possibility of an acute increase in IAP and, if not already done, should result in immediate IAP quantification.

Recently, study results from various pilot projects and model validations of novel IAP measurement methods have been published [27,28,29,30]. Unfortunately, the majority of these methods have not even found their way out of preclinical testing into the clinical routine of adult medicine—let alone pediatrics. Even if there are advantages due to their non-invasiveness and continuous data collection, there are clear disadvantages in test series so far [27], such as

- lack of accuracy, sensitivity and reliability
- high susceptibility to errors (e.g., due to movement artefacts and sensitivity)
- lack of standardization or even lack of proof of concept
- high costs.

A further development of bladder pressure measurement via the Foley catheter could be the most promising new development, enabling the continuous and automated IAP assessment with the help of the novel so-called “SERENNO” equipment [30]. Apart from such conventional indirect pressure measurements via abdominal or retroperitoneal hollow organs, the microwave-based “transient radar method” seems to have the necessary potential, on the basis of which a translation into clinical routine could appear possible. However, due to the special anatomical and pathophysiological conditions in children and especially in premature and newborn infants, as well as the associated vulnerability, testing in pediatrics will only be conceivable once it has been successfully validated and established in adult medicine. 

### 4.4. Study limitations

We pooled all the matched longitudinal measures used for the primary and the explorative assessments, ignoring the circumstance that some of them came from the same patients. We judge this to be feasible because the states of these subjects during their stay in ICU differed substantially in terms of hemodynamics, respiration, vigilance and several additional determinants. The variability resulting from these changing state combinations is likely to cause more influences than the fact that data sets were in part from identical subjects.

In sum, the findings of the primary and exploratory analyses did not reveal any important or significant difference. Therefore, targeted as well as not-targeted influences could not affect the poor measuring agreement of the tested FVP and IVP or IGP measurement methods.

**Table 5:** Literature overview of publications concerning FVP measurements.

Study Type/Model			Author Year [Ref.]	Measure Methods	Methods–Statistics	No of Subjects Enrolled (No. of Paired Measures)	IAP Range (mmHg)	Comparisons	Results					Conclusion
									Correlation Coefficient	WSACS Method Validation Criteria				
										Bias (mmHg)	Precision (mmHg)	LOA (mmHg)	PE (%)	
in-vivo	Animals		Lacey 1987 [22]	IVCP (FVP) IVP IGP	Correlation coefficient (unspecified)	17 rabbits (n.a.)	0–30	IAP versus IVCP (FVP) IAP versus IVP IAP versus IGP	>0.87 >0.85 0.7	n.a.	n.a.	n.a.	n.a.	FVP usable
			Gudmundsson 2002 [11]	FVP IVCP IVP	Correlation coefficient (unspecified)	8 pigs (n.a.)	15–40	IAP versus FVP IAP versus IVCP IAP versus IVP	0.95 0.94 0.92	n.a.	n.a.	n.a.	n.a.	FVP usable
			Jakob 2010 [23]	IGP IVP IVCP (FVP)	Pearson correlation coefficient (r²) Bias LOA	12 pigs (n.a.)	0–22	IGP versus IVP IGP versus IVCP IVP versus IVCP	0.60 0.63 0.52	n.a.	n.a.	n.a.	n.a.	FVP limited usable
			Regli 2010 [24]	FVP IVP	Correlation coefficient (r²) Bias, precision	13 pigs (n.a.)	3–26	IVP versus FVP	0.89	5.0	3.8	n.a.	n.a.	FVP limited usable
	Human	Adults	Joynt 1996 [12]	SVCP IVCP (FVP)	Bias precision LOA	19 (133)	1–26	SVCP versus IVCP	n.a.	0.45	0.89	−1.33 to 2.23	n.a.	FVP usable
			Ho 1998 [13]	SVCP IVCP	Bias, precision LOA	20 (140)	n.a.	SVCP versus IVCP	n.a.	0.1	1.06	−2.04 to 2.2	n.a.	FVP usable
			Markou 2004 [25]	IVCP (FVP) IVP	Pearson correlation coefficient	38 (151)	n.a.	IVP versus IVCP 1. IAP < 10 mmHg 2. IAP 10–15 mmHg 3. IAP > 15 mmHg	0.76 0.69 0.78	n.a.	n.a.	n.a.	n.a.	FVP not usable
			De Keulenaer 2011 [26]	IVP FVP	Bias, precision LOA	149 (866)	6.7–22.4	IVP versus FVP 1. pooled IAP 2. IAP ≥ 12mmHg 2. IAP > 20 mmHg	n.a.	−1.5 0.4 0.7	3.6 3.9 2	−8.6 to 5.7 −8.1 to 7.3 −3 to 4.6	n.a.	FVP not usable
			Howard 2016 [31]	IVP FVP	All WSACS method validation criteria	11 (53)	0–25	IVP versus FVP pooled without weight artificially increased IAP 5 kg artificially increased IAP 10 kg	0.8 n.a. n.a. n.a.	2.8 3.2 2.5 2.5	3.42 3.63 3.92 2.26	−4.1 to 9.6 −4.1 to 10.4 −5.4 to 10.3 −2.1 to 7	46.8 n.a. n.a. 27.1	FVP not usable
		Children	Present study	FVP IVP IGP	All WSACS method validation criteria	39 (1119)	1–23	FVP versus IVP FVP versus IGP	0.14 0.13	0.5 −0.8	4.2 4.4	−7.9 to 8.9 −9.6 to 8.0	133 117	FVP not usable

The publications were subdivided according to study models. The last column contains the conclusion regarding the usefulness of the FVP method (color-coded according to the traffic light system). Abbreviations: FVP: femoral vein pressure; IAP: intra-abdominal pressure; IGP: intra-gastric pressure; IVCP: inferior vena cava pressure; IVP: intra-vesical pressure (bladder pressure); r^2^ degree of certainty (correlation coefficient); SVCP: superior vena cava pressure; bias: mean difference between 2 measures; precision: SD of the bias; LOA: limits of agreement; percentage error: Quotient of LOA and mean IAP.

## 5. Conclusions

Our data suggest that FVP does not reliably reflect IAP and that its use in clinical practice can lead to a fatal misjudgment in critically ill children. For the time being, IAP measurements in children should therefore continue to be made by quantifying IVP or IGP, without exception.

## Figures and Tables

**Table 1:** Amended * WSACS guidelines regarding the minimum requirements for the exchangeability of two competing IAP assessment techniques.

	No. of Enrolled Patients	r^2^ *	Bias [mmHg]	Precision [mmHg]	LOA [mmHg]	Percentage Error [%]
Target values	≥20	≥0.6 *	≤|1|	≤2	−4 to +4	≤25

* The original WSACS recommendations do not consider a correlation coefficient (r^2^) as a parameter for assessing interchangeability. However, an r^2^ of at least 0.6 should be assumed. Abbreviations and definitions: FVP: femoral vein pressure; IGP intra-gastric pressure; IVP: intra-vesical pressure (bladder pressure); r^2^: Spearman’s correlation coefficient; bias: mean difference between two measures; precision: SD of the bias; LOA: limits of agreement (=upper and lower SD of the mean bias); percentage error: quotient of LOA and mean IAP.

**Table 2:** Description of the patient population (presented as mean ± standard deviation [SD]).

Parameter	FVP vs. IVP	FVP vs. IVP
Number of patients	19	20
Number of girls	6	7
Number of paired measures	459	660
Age [years]	4.9 ± 5.3	4.7 ± 5.3
	Newborn [*n* = 1]	Newborn [*n* = 2]
	Sucklers [*n* = 5]	Sucklers [*n* = 5]
	Tots [*n* = 6]	Tots [*n* = 6]
	School [*n* = 5]	Schoolkids [*n* = 5]
	Teenagers [*n* = 2]	Teenagers [*n* = 2]
Body mass index *	16.7 ± 4.2	16.7 ± 4.1
Duration of the stay at PICU [days]	24.3 ± 41.6	21.8 ± 38.9
Admission-justifying diagnoses (responsible clinical department)	Ped. Cardiology [*n* = 4]	Ped. Cardiology [*n* = 6]
	Neurosurgery [*n* = 3]	Neurosurgery [*n* = 3]
	Pedosurgery [*n* = 5]	Pedosurgery [*n* = 4]
	Ped. pulmonology [*n* = 1]	Ped. pulmonology [*n* = 1]
	Ped. traumatology [*n* = 6]	Ped. traumatology [*n* = 6]
PRISM-III-score		
- First day of enrolment	11.4 ± 6.5	11.3 ± 6.5
- Last day of enrolment	2.6 ± 2.8	2.6 ± 2.7 *
Lethality	0%	5% [1 deceased] **

* Definition: body mass index BMI = kg/m^2^. PICU: Pediatric intensive care unit; PRISM III Score: Pediatric Risk of Mortality Score III. ** The dataset of the child who died was not taken into account when assessing the PRISM score on the final day of trial participation.

**Table 3:** Findings of the comparative statistics analyzing the FVP and IGP measurement agreement.

		No. of Measurements	IGP [mmHg]	FVP [mmHg]	r^2^	Bias [mmHg]	Precision [mmHg]	LOA [mmHg]	Percentage Error [%]	Mean Absolute Percentage Error (SD) [%]
			Median [Range]							
Overall Result		660	7.0 (2.4–16.5)	7.0 (1.0–23.0)	0.13	−0.8	4.4	−9.6–8.0	117	55 (47)
Explorative additional examination: influenceability by right cardiac valve insufficiency										
Echo- findings	No TI, no PI and no PHT	48	5.7 (4.0–10.9)	3.0 (1.0–13.0)	0.13	2.1	2.3	−2.5 to 6.7	90	41 (29)
	Mild TI, mild PI and no PHT	271	7.0 (2.5–16.5)	10.0 (1.0–23.0)	0.10	−2.2	4.2	−10.6 to 6.2	102	62 (50)
	Mild to moderate TI and/or PI and/or PHT	120	6.8 (3.5–12.9)	10.0 (1.0–19.0)	0.47	−1.8	3.6	−9.0 to 5.4	91	50 (27)
	No Echo findings available	221	7.1 (2.4–14.2)	5.0 (1.0–21.0)	0.06	0.8	4.6	−8.4 to 10.0	135	52 (53)
Exploratory additional examination: influenceability by the height of intra-abdominal pressure (IAP)										
IAP [mmHg]	<7	189	5.2 (2.4–6.9)	3.0 (1.0–6.0)	0.01	1.7	1.9	−2.1 to 5.5	88	42 (25)
	7–9	189	7.8 (3.5–9.8)	7.0 (1.0–9.0)	0.08	1.6	3.1	−4.6 to 7.8	93	40 (27)
	10–12	162	7.8 (2.7–12.6)	11.0 (2.0–12.0)	0.15	−2.3	3.6	−9.5 to 4.9	80	57 (43)
	13–15	83	8.3 (3.9–14.2)	14.0 (7.0–15.0)	0.02	−5.2	2.7	−10.6 to 0.2	49	76 (49)
	16–18	23	8.4 (3.7–12.9)	17.0 (16.0–18.0)	0.05	−8.5	2.9	−14.3 to −2.7	46	123 (80)
	>16	14	8.0 (3.8–16.5)	19.0 (19.0–23.0)	0.05	−11.5	3.2	−17.9 to −5.3	46	170 (99)

Abbreviations: FVP: femoral vein pressure; IGP: intra-gastric pressure; r^2^: Spearman’s correlation coefficient; mean bias: difference between 2 measurements; precision: SD of the mean bias; LOA: limits of agreement; percentage error: Quotient of LOA and mean IAP; Echo: Echocardiography; TI: tricuspid regurgitation; PI: pulmonary regurgitation; PHT: pulmonary hypertonia; IAP intra-abdominal pressure.

**Table 4:** Findings of the comparative statistics analyzing the FVP and IVP measurement agreement.

		No. of Measurements	IVP [mmHg]	FVP [mmHg]	r^2^	Bias [mmHg]	Precision [mmHg]	LOA [mmHg]	Percentage Error [%]	Mean Absolute Percentage Error (SD) [%]
			Median [Range]							
Overall Result		459	6.0 (1.0–16.0)	5.0 (1.0–22.0)	0.14	0.5	4.2	−7.9 to 8.9	133	51 (55)
Exploratory additional examination: influenceability by right cardiac valve insufficiency										
Echo- findings	No TI, no PI and no PHT	48	5.0 (3.0–12.0)	3.0 (1.0–13.0)	0.02	1.5	2.6	−3.7 to 6.7	108	37 (32)
	Mild TI, mild PI and no PHT	117	6.0 (1.0–16.0)	5.0 (1.0–22.0)	0.30	−0.4	4.0	−8.4 to 7.6	123	56 (60)
	Mild to moderate TI and/or PI and/or PHT	73	6.0 (2.0–11.0)	6.0 (1.0–19.0)	0.48	−0.7	3.3	−7.3 to 5.9	103	46 (35)
	No Echo findings available	221	6.0 (3.0–14.0)	5.0 (1.0–21.0)	0.04	0.2	4.7	−9.2 to 9.6	145	54 (61)
Exploratory additional examination influenceability by the height of intra-abdominal pressure (IAP)										
IAP [mmHg]	<7	203	5.0 (1.0–6.0)	3.0 (1.0–6.0)	0.06	1.3	1.8	−2.3 to 4.9	87	38 (42)
	7–9	153	7.0 (3.0–9.0)	6.0 (1.0–9.0)	0.08	1.6	3.0	−4.4 to 7.6	94	30 (40)
	10–12	49	8.0 (3.0–12.0)	10.0 (2.0–12.0)	0.45	−1.1	4.7	−10.5 to 8.3	107	64 (62)
	13–15	27	8.0 (4.0–15.0)	14.0 (12.0–15.0)	0.01	−5.0	2.8	−10.6 to 0.6	50	73 (50)
	16–18	16	8.0 (4.0–9.0)	17.0 (16.0–18.0)	0.00	−9.9	1.8	−13.4 to −6.3	30	152 (73)
	>16	11	6.0 (4.0–16.0)	19.0 (19.0–22.0)	0.68	−12.2	2.5	−17.2 to −6.7	37	199 (93)

Abbreviations: FVP: femoral vein pressure; IVP: intra-vesical pressure (bladder pressure); r^2^: Spearman’s correlation coefficient; mean bias: difference between 2 measurements; precision: SD of the mean bias; LOA: limits of agreement; percentage error: Quotient of LOA and mean IAP; Echo: Echocardiography; TI: tricuspid regurgitation; PI: pulmonary regurgitation; PHT: pulmonary hypertonia; IAP intra-abdominal pressure.

## Data Availability

Raw data and research findings can be obtained at the authors’ premises upon justified demand.

## Abbreviations

ACM	Air capsule-based measurement
ACS	Abdominal compartment syndrome
BMI	Body mass index
FVP	Femoral vein pressure
IAH	Intra-abdominal hypertension
IAP	Intra-abdominal pressure
IGP	intra-gastric pressure
IVCP	Inferior Vena cava pressure
IVP	Intra-vesical pressure
LA	Limits of agreement
LOS-PICU	Duration of stay at PICU
MHH	Medizinische Hochschule Hannover
PE	Percentage error
PHT	Pulmonary hypertension
PI	Pulmonary valve insufficiency
PICU	Pediatric intensive care unit
PRISM-III	Pediatric risk of mortality score III
SD	Standard deviation
SVCP	Superior vena cava pressure
TI	Tricuspid valve insufficiency
WHO	World Health Organisation
WSACS	Abdominal Compartment Society (formerly known as World Society of Abdominal Compartment Syndrome)
